# *Cydonia oblonga* Mill. Pulp Callus Inhibits Oxidative Stress and Inflammation in Injured Cells

**DOI:** 10.3390/antiox12051076

**Published:** 2023-05-10

**Authors:** Federica Gubitosa, Daniele Fraternale, Roberta De Bellis, Andrea Gorassini, Leila Benayada, Laura Chiarantini, Maria Cristina Albertini, Lucia Potenza

**Affiliations:** 1 Department of Biomolecular Sciences, University of Urbino Carlo Bo, 61029 Urbino, Italy; 2 Department of Humanities and Cultural Heritage, University of Udine, 33100 Udine, Italy; andrea.gorassini@uniud.it

**Keywords:** RAW 264.7, HaCaT cell, anti-inflammatory, antioxidant, quince, callus culture

## Abstract

The pharmacological activity of a callus extract from the pulp of *Cydonia oblonga* Mill., also known as quince, was investigated in murine macrophage (RAW 264.7) and human keratinocyte (HaCaT) cell lines. In particular, the anti-inflammatory activity of *C. oblonga* Mill. pulp callus extract was assessed in lipopolysaccharides (LPS)-treated RAW 264.7 by the Griess test and in LPS-treated HaCaT human keratinocytes by examining the expression of genes involved in the inflammatory process, including nitric oxide synthase (iNOS), interleukin-6 (IL-6), interleukin-1β (IL-1β), nuclear factor-kappa-B inhibitor alfa (ikBα), and intercellular adhesion molecule (ICAM). The antioxidant activity was evaluated by quantizing the reactive oxygen species (ROS) production in the hydrogen peroxide and tert-butyl hydroperoxide-injured HaCaT cell line. The obtained results indicate that *C. oblonga* callus from fruit pulp extract has anti-inflammatory and antioxidant activities, suggesting its possible application in delaying and preventing acute or chronic diseases associated with aging or in the treatment of wound dressing.

## 1. Introduction

*Cydonia oblonga* Mill. (quince) is a very old, spontaneous, and cultivated fruit tree, originating in Asia Minor, around 200 BC, when it was cultivated by the Greeks and Babylonians. Today, quince trees are widespread mainly in the western Mediterranean areas and China. In Italy, it is cultivated in all regions and appears in a sub-spontaneous state at about 1500 m above sea level. The plant flowers in spring, between April and May. Its fruit is a subglobose or pear-shaped apple with a bright yellow color, an astringent taste, and a characteristic aroma. It is possible to identify two varieties of quince: The first one has apple-shaped fruits, and the second one has pear-shaped fruits. The two varieties also differ in taste, with the apple-shaped variety having a harder pulp and a more astringent taste than the pear-shaped variety [1].

Plants, including quince, are a valuable source of chemical compounds, called secondary metabolites (SMs), which are frequently used as the primary source in the development of food additives, functional foods, and new drugs thanks to their ability to exert several activities useful for human health, such as antibacterial, antiviral, anti-tumoral, antioxidant, anticonvulsant, analgesic, anti-inflammatory, and antidepressant effects [1,2]. These molecules are produced in many varieties of plants, but their natural production is modest and un-homogeneous due to a variety of factors, such as season, environment, climate, and cultivar, which make their extraction from plant matrices difficult and require the use of solvent, steam, and supercritical fluids [3]. At the same time, for SMs production, it is necessary to resort to chemical synthesis or the use of large quantities of water and fruit material. In vitro propagation techniques, using plant cell cultures, have been developed to minimize these disadvantages, and the most employed are based on the formation of callus cultures from adult plant cells. Therefore, callus from hairy, root, organ, or pulp-controlled cultures in vitro must be preferred to obtain valuable SMs and reproducible, good quality products, regardless of seasonal and geographical factors faced by a smaller amount of water used and the lower production of waste, according to the Green Economy principles [4].

Our previous study described the production of callus culture extract obtained from the pulp of *Cydonia oblonga* Mill. (from now on *Co ext*.), the hydroalcoholic extract preparation, the chemical composition in SMs, such as phenolic and triterpenic acid, and the biological activity in cell-free models [5].

Taking these preliminary results into account, in the present study, we moved on to evaluate the anti-inflammatory activity of *Co ext.* in LPS-stimulated RAW 264.7 cells, by analyzing the NO production, and in LPS-stimulated HaCaT cells, by evaluating the expression of pro-inflammatory genes (iNOS, IL-6, IL-1β, ikBα, and ICAM). Furthermore, we measured the ROS production in the hydrogen peroxide and tert-butyl hydroperoxide-injured HaCaT cell line.

## 2. Materials and Methods

### 2.1. Callus Production

*C. oblonga* ripe fruits were collected from an orchard located in Urbino (PU), Marche region, Italy. The apples were opened with a sterile blade under the laminar flow cabinet, after surface sterilization with ethanol 90%. Cultures were then conducted as previously described by our team [5]. Briefly, the combination of Gamborg B5 medium plus 1.77 µM BA (6-benzylaminopurine, Sigma-Aldrich) and 5.40 µM NAA (1-Naphthaleneacetic acid, Sigma-Aldrich, Milan, Italy), 30 g/L sucrose, and pH 5.8 induced the highest biomass production from pulp explants of *C. oblonga* fruits. Cultures were incubated in the dark at 25 ± 2 °C, and subcultures were obtained after 28 days in the same media. The callus obtained by subcultures was stored at −80 °C and lyophilized before the extraction.

### 2.2. Callus Extract Preparation

Callus extract from *C. oblonga* (*Co ext.*) was prepared following the previously described protocols [5,6]. Briefly, 130 mg of callus freeze-dried was homogenized in a Potter-type homogenizer. The dried material was dissolved in 15 mL of diluted ethanol solution (70%) and shaken overnight (ON). The mixture was centrifuged at 13,000 rpm for 45 min at 4 °C, and the supernatant was recovered, filtered with 0.2 µm filters, aliquoted, dried, and weighed (dry weight—dw). Before use, each aliquot was dissolved in double distilled water at 30 mg dw/mL.

### 2.3. Chemical Characterization of SMs in Quince Callus Extract

Characterization and quantification of triterpenic acids and polyphenolic compounds in *Co ext.* were performed by GC–MS analyses and HPLC-DAD-ESI-MSn, respectively, as reported by De Bellis et al. [5].

### 2.4. Cell Culture

HaCaT (immortalized human keratinocytes) and murine RAW 264.7 macrophages were acquired from CLS-Cell Lines Service GmbH (Eppelheim, Germany) and from the European Collection of Cell Cultures (Salisbury, UK), respectively. Cell lines were grown in Dulbecco’s Modified Eagle’s Medium (DMEM, Sigma-Aldrich, Milan, Italy) completed with 1 mM pyruvate, 100 mM non-essential amino acids, 10% (*v*/*v*) heat-inactivated fetal bovine serum (FBS), penicillin (100 U/mL), and streptomycin (100 μg/mL), 1% (*w*/*v*) L glutamine. The cells were incubated under standard conditions (37 °C, 5% CO_2_, 95% humidity). The growth medium was changed every 2–3 days till approximately 80% confluence was reached. All experiments were performed after a 24 h period of cell adhesion.

### 2.5. Cell Viability Assay WST8

Cell viability assay was performed according to Tiboni et al. [7]. HaCaT and RAW 264.7 cells were seeded into 96-well plates (5 × 10^3^ cells per well) in 100 µL complete DMEM and incubated at 37 °C for 24 h. The medium was then removed, and 100 µL of fresh medium containing *Co ext.* at increasing concentrations (0–4 mg/mL) were added and incubated for a further 24 h. After 24 h, 10 µL of water-soluble tetrazolium salt (WST-8) (Sigma-Aldrich, Milan, Italy) were added to each well to evaluate cell metabolic activity. Absorbance was measured at 450 nm using a plate reader (BioRad Laboratories, Hercules, CA, USA) up to a maximum of 4 h of incubation at 37 °C. Three independent experiments were performed in triplicate, and cell viability was assessed as a percentage of the control.

### 2.6. Nitric Oxide Detection

RAW 264.7 cells stimulated by lipopolysaccharide (LPS, Sigma-Aldrich, Milan, Italy) were used to evaluate anti-inflammatory properties. Determination of extracellular nitric oxide (NO) concentration was carried out by UV-visible spectrophotometer. The nitrite concentration was determined according to a linear standard curve (calcium nitrite 6.25–100 µM) [8].

RAW 264.7 cells, in the logarithmic phase, (3 × 10^4^ cells/100µL) were seeded in 96-well plates and cultured for 24 h. Then, cells were co-treated for 24 h with LPS (1 µg/mL) in the presence or absence of *Co ext.* 0–4 mg/mL. Dexamethasone (DEXA) 0.0039 mg/mL was used as positive control. After 24 h, aliquots of 50 µL of supernatant were taken and incubated for 10 min in the dark at room temperature with 50 µL of Griess reagent at a concentration of 40 mg/mL. Absorbance was measured at 570 nm using a plate reader (BioRad Laboratories, Hercules, CA, USA).

To exclude the possibility that the reduction in NO could be linked to a reduction in cell viability, it was assessed in the same plate using the WST-8 viability assay.

### 2.7. LPS-Induced Inflammation in HaCaT Cells

Inflammation on HaCaT cells was induced by 1 µg/mL LPS [9]. HaCaT cells, in the logarithmic phase, were seeded at a density of 6 × 10^5^ cells/mL (2 mL/well) on 6-well plates and allowed to adhere for 24 h in a 37 °C incubator with 5% CO_2_. Then, the cells were co-treated for 24 h with LPS in the presence or absence of 1.78 mg/mL *Co ext.* (value derived from a previous viability study). At the end of the treatment, the medium was removed, and the cells were washed in PBS and trypsinized. The cells were stored in 350 µL of lysis buffer RLT (RNeasy mini kit Qiagen, Milan, Italy) at −80 °C until RNA extraction.

### 2.8. Quantitative Real-Time PCR

Total RNA was extracted using the RNeasy mini kit Qiagen, Milan, Italy, and then mRNA was converted to cDNA and processed by real-time PCR to evaluate the expression of iNOS, IL-6, IL-1β, ikBα, and ICAM genes related to the inflammatory process. The expression levels of the inflammatory genes were normalized with the housekeeping gene 36B4. Quantification was performed in a 20 µL volume using 2 µL of each cDNA. Specifically, 10 µL of Power up Sybr Green Master Mix, 0.6 µL of forward and reverse primers from a 10 µM preparation, and 6.8 µL of water were used. Real-time PCR was conducted using a QuantStudio 1 Real-Time PCR System thermal cycler (Thermo Fisher). The analysis was carried out with the Pfaffl method [10]. The real-time PCR thermal conditions were carried out as follows: initial denaturation at 95 °C for 10 min, and then 40 cycles at 95 °C for 15 sec and 60 °C for 1 min.

The nucleotide sequences of the primers are summarized in Table 1.

### 2.9. Quantification of Intracellular ROS Production

Intracellular ROS (reactive oxygen species) was determined by FLUOstar OPTIMA fluorescence microplate reader (BMG LABTECH) using the cell-permeable fluorogenic probe 2′,7′ -dichlorofluorescein diacetate (DCF-DA). HaCaT cells were seeded into a 96-well plate at a density of 1 × 10^4^ cells in 100 µL of growth medium for 48 h at 37 °C, then washed with PBS, and treated with different concentrations of *Co ext.* in DMEM without FBS and antibiotics for 2 h at 37 °C. After the treatment, the cells were washed with PBS, and then 10 μM DCF-DA was added for 30 min. The DCF-DA was removed, by washing with PBS, and ROS production was determined. A total of 0.1 mM H_2_O_2_ or 0.3 mM *tert*-butyl hydroperoxide (TBHP) was added, and after 30 min of treatment, ROS production was determined again.

### 2.10. Statistical Analysis

The statistical analyses were performed using GraphPad Prism version 6 for Windows, GraphPad Software. Values were expressed as the mean ± the standard error of the mean (SEM). The treated and control sample variables were compared using one-way analysis of variance (ANOVA) followed by Dunnett’s post hoc test. The differences between samples were considered significant with *p*-values < 0.05.

## 3. Results

### 3.1. Effects of Co ext. on Cell Viability

**Figure 1:**
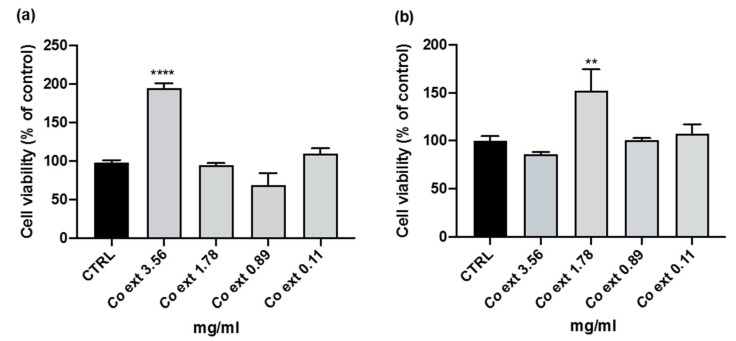
RAW 264.7 and HaCaT cell viability. RAW 264.7 (**a**) and HaCaT (**b**) cells were treated with different concentrations of *Co ext.* (0–4 mg/mL) for 24 h, and cell viability was measured by WST-8 assay. Data are the means of three independent experiments conducted in triplicate ± SEM. (** *p* < 0.01, **** *p* < 0.0001) compared to CTRL (untreated cells).

The non-cytotoxic concentration range was identified in both RAW 264.7 and HaCaT cell lines, after 24 h of *Co ext.* treatment by cell viability WST-8 assay. The analyses showed that none of the tested concentrations were cytotoxic, with even a significant increase in cell viability at the *Co ext.* concentration of 3.56 mg/mL and 1.78 mg/mL in RAW 264.7 and HaCaT cells, respectively (Figure 1).

### 3.2. Anti-Inflammatory Activity of Co ext.

To determine the *Co ext.* anti-inflammatory properties, we evaluated the LPS-induced NO release in murine macrophages RAW 264.7 cells.

**Figure 2:**
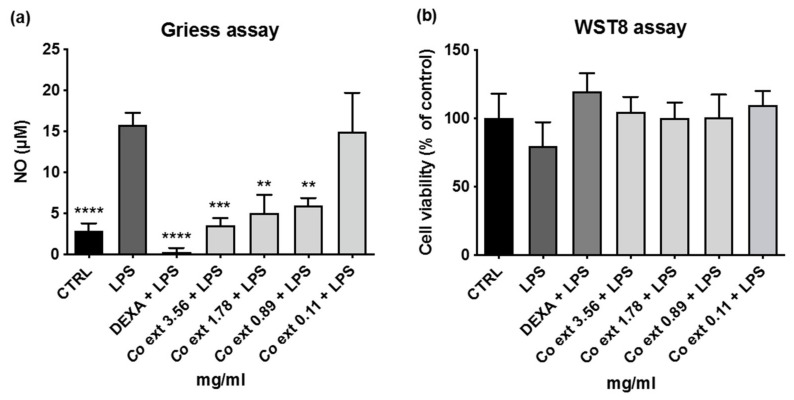
Extracellular NO release and cell viability evaluation after LPS, *Co ext*., and dexamethasone (DEXA) administration to RAW 264.7 cells. (**a**) RAW 264.7 cells were co-treated for 24 h with LPS 0.001 mg/mL in the presence or absence of *Co ext.* or DEXA. CTRL: negative control (untreated cells); DEXA 0.0039 mg/mL was used as an anti-inflammatory reference compound. The results are presented as nitric oxide concentration. The data show the means ± SEM of all samples compared to LPS (** *p* < 0.01, *** *p* < 0.001, **** *p* < 0.0001). Dunnett’s multiple comparison test was performed. (**b**) Evaluation of cell availability (WST8 assay). The results are expressed as the percentage of control ± SEM of three independent measurements.

The co-treatment of RAW 264.7 cells for 24 h with 1 µg/mL LPS and *Co ext.* (0–4 mg/mL) showed a significant reduction in NO release at all tested concentrations except for 0.11 mg/mL *Co ext.* (Figure 2a). To exclude the possibility that the NO reduction could be linked to a reduction in cell viability after LPS administration the WST-8 assay was also performed. As shown in the figure, none of the *Co ext.* concentrations tested led to a reduction in cell viability (Figure 2b).

**Figure 3:**
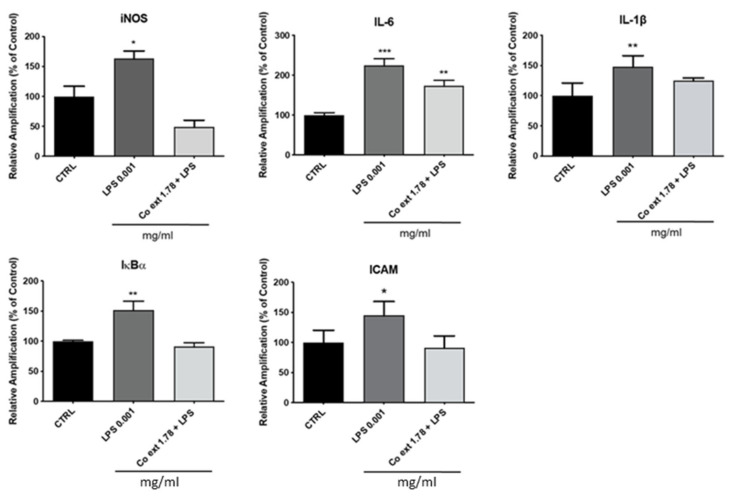
Anti-inflammatory activity of *Co ext.* on LPS-treated HaCaT cells. HaCaT cells were co-treated for 24 h with LPS 0.001 mg/mL in the presence or absence of 1.78 mg/mL *Co ext. Co ext.* significantly decreased the expression of all the LPS-upregulated analyzed genes. Data are the means ± SEM of three independent experiments. (* *p* < 0.1, ** *p* < 0.01, *** *p* < 0.001). Dunnett’s multiple comparison test was performed vs. CTRL.

The anti-inflammatory effect of *Co ext.* on HaCaT cells was evaluated after the co-treatment for 24 h with 1 µg/mL LPS in the presence or absence of the extract by investigating the expression of iNOS, IL-6, IL-1β, ikBα, and ICAM by real-time PCR. These genes, linked to the inflammatory process, were downregulated by the co-treatment with 1.78 mg/mL *Co ext.* (Figure 3).

### 3.3. Effects of Co ext. on Antioxidant Activity

**Figure 4:**
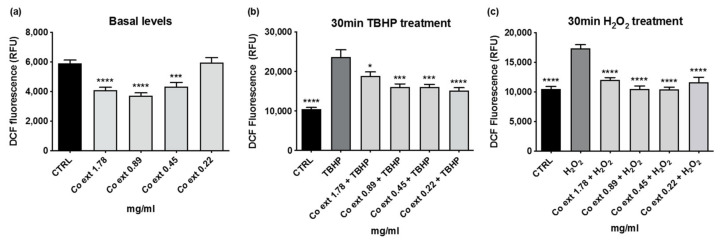
Antioxidant activity of *Co ext.* in HaCaT cells. Intracellular ROS were evaluated using the DCF-DA assay in HaCaT cells treated with different concentrations of *Co ext.* ROS production was detected (**a**) after ON incubation with 0 (=CTRL), 1.78; 0.89; 0.45; 0.22 mg/mL *Co ext.* without any oxidative stimulus and (**b**) after treatment with 0.3 mM TBHP for 30 min or (**c**) 0.1 mM H_2_O_2_ for 30 min. Dunnett’s multiple comparison test was performed vs. CTRL (**a**), TBHP (**b**), H_2_O_2_ (**c**). Data represent the mean ± SEM of at least three independent experiments. (* *p* < 0.05; *** *p* < 0.001; **** *p* < 0.0001).

Preliminary studies were also carried out to evaluate *Co ext.* antioxidant properties. Due to the presence of polyphenols in *Co ext.* [5] and to the relevance of these compounds for human health, the antioxidant activity of *Co ext.* was investigated on HaCaT cells using the fluorescent probe DCF-DA. Using this probe, we found that the ROS production was significantly decreased by *Co ext.* already in the absence of an oxidative challenge (Figure 4a). The ROS production induced by 0.3 mM tert-butyl hydroperoxide (TBHP) or by 0.1 mM H_2_O_2_ for 30 min was significantly reduced by *Co ext.* (Figure 4b,c).

## 4. Discussion

This study aimed to investigate the biological activities, particularly the anti-inflammatory and antioxidant properties, of the set of SMs produced by the callus obtained from the pulp of *C. oblonga* Mill. on two different cellular models (RAW 264.7 and HaCaT). The set of active molecules naturally present in medicinal plants is called the phytochemical complex. In plants, these substances can act individually and/or in concert. Often, when the various phytocomplex constituents are isolated, their biological activities are minimal, while the effects of the whole phytocomplex as it is in the plant are remarkable [11]. Dietary intake of phytochemicals with nutraceutical properties promotes health benefits and protects the body against numerous diseases, including cancers, coronary heart disease, diabetes, high blood pressure, inflammation, microbial viral and parasitic infections, psychotic disease, spasmodic conditions, ulcers, and osteoporosis [12]. Furthermore, phytochemicals may represent a new nutraceutical therapeutic approach to preventing or mitigating diet-related diseases, which are becoming more common in Western societies because of the increased availability of calorie-rich foods and a sedentary lifestyle [13]. Obesity, diabetes, atherosclerosis, and neurodegeneration are the most common diseases associated with incorrect diet and are all characterized by low-grade inflammation underlying the disease. Nutraceuticals are plant-based or food-derived products containing physiologically beneficial substances that can be used to improve health and prevent chronic disease. In 1989, Stephen De Felice coined the term “nutraceutical” referring to this term as “a food (or part of a food) that provides medical or health benefits, including disease prevention and/or treatment”. Nutraceuticals may, therefore, be designed not only as food supplements but also as useful tools for the prevention and treatment of diseases and/or disorders [14]. Several studies are currently being conducted using plant cell cultures to achieve SMs that can be used as nutraceuticals [5,6,15,16]. For several years, the long-term bioactive compound exploitation has required the implementation of biotechnological strategies for high functional value and economically viable products. In this field, callus cultures have become a promising strategy for the biosynthesis of valuable SMs in a limited amount of time and space [17]. This approach has been suggested as an alternative method to produce plant SMs, which in fresh fruits or in other parts of the plant are present in lower concentrations, and to avoid the limitations due to the season and plant reproductive cycle [15]. Apple consumption has been linked to a lower risk of certain cancers, cardiovascular and respiratory diseases, and diabetes in epidemiological studies. It has recently been discovered that apples have beneficial effects on vascular function, acting as a modulator of blood pressure, plasma lipid levels, inflammation, and hyperglycemia. The presence of bioactive compounds in the apple prompted the researchers to set up callus cultures from this fruit [18]. Oota was the first to implement apple callus culture and found a high content of anthocyanins [19]. Recently, Verardo et al. isolated callus from Golden Delicious, “Mela Rosa Marchigiana”, and the exotic Acca Sellowiana (Berg.) Burret ripe pulps; all these derived callus cultures were richer in triterpenic acids than the respective peels and pulps [15,16]. Additionally, quince, a widely available and low-cost food source of beneficial phytoconstituents, has been investigated as a potential starting material to produce nutraceuticals [20], and more recently, we obtained SMs using pulp callus culture [5]. In the same study, the chemical characterization of phenolic and triterpenic acid contents of *Co ext.* and its biological activity in cell-free models were reported. Briefly, the total phenolic content of callus extract was 8.3 times higher than that of the pulp. The most represented phenolic compound in callus extract was 5-CQA followed by 5-p-coumaroylquinic acid (5-p-CoQA), and 3-O-caffeoylquinic acid (3-CQA). The compounds 3-CQA and procyanidin B2 were found to be more expressed in the pulp than in the callus. The main triterpenic acids present in the callus were maslinic, corosolic, annurcoic, and tormentic acids, while they were completely absent in the pulp. Other studies have been conducted on the phytochemical composition of quince focusing mainly on the polyphenolic profile of the various parts of the fruit [2,20,21]. No studies concerning the triterpene acids profile of quince are reported in the literature, thus our team was the first to develop quince pulp callus cultures and to characterize the triterpene acids and polyphenols contents [5]. This background and the known antioxidant and anti-inflammatory properties of polyphenols and triterpenic acids [22,23,24] prompted us to investigate the *Co ext.* effects in cellular models. Macrophages have an important role in immune reactions, allergies, and inflammation protecting the body from foreign pathogens through phagocytosis. During this process, macrophages release a variety of inflammatory mediators, including interleukin 1 (IL-1), tumor necrosis factor-alpha (TNF-α), nitric oxide (NO), and prostaglandins [25]. LPS-activated macrophages produce nitric oxide synthase (iNOS), an enzyme that catalyzes the oxidative deamination of L-arginine to produce NO. Excess NO production by iNOS can have negative consequences, such as inflammatory diseases and septic shock [25]. NO released by macrophages stimulated with LPS is a marker associated with acute and chronic inflammation processes [26]. We selected the RAW 264.7 cells as an appropriate macrophage model to rapidly study the inflammatory cell responses with the Griess test [27]. In this model, our findings reveal that *Co ext*. can be safely used and does not have any cytotoxic effects on the RAW 264.7 cell line. Moreover, *Co ext*. has a strong anti-inflammatory activity by being able to reduce the release of NO during the inflammatory process induced by LPS.

As a human cell model, we chose the keratinocyte cell lines (HaCaT) because they are a cellular model that allows us to study epidermal homeostasis and its pathophysiology.

Keratinocytes are primarily responsible for the structural and barrier functions of the epidermis; they are also involved in activating and maintaining inflammatory and immunological responses in the skin, and they actively participate in wound repair through the secretion of growth factors, cytokines, and chemokines [28]. Given the importance of the function of the skin barrier, the effect of *Co ext.* was investigated in HaCaT cells also in view of our future studies aimed at evaluating the effect of the extract in the treatment of skin-related diseases. In this cellular model, we evaluated how *Co ext.* can modulate the expression of pro-inflammatory cytokines, and we found that *Co ext.* is able to downregulate the expression of genes related to the inflammatory process. Moreover, although a more in-depth investigation will be required, the downregulation of the selected cytokines suggests that *Co ext.* acts through the involvement of the NF-κB signal transduction pathway.

In HaCaT cells, we also found that *Co ext.* does not have a toxic effect on all doses tested and can act as an antioxidant keeping the production of ROS inside the cells under control. These results confirmed also in a cellular model the antioxidant activity of *Co ext.*, previously observed in cell-free models detected by DPPH, ABTS, and ORAC assays [5]. *Co ext.* is very rich in polyphenols as previously reported [5], and these compounds are powerful antioxidants that can prevent or slow down the oxidation of free radicals [29]. They can scavenge a wide range of reactive oxygen species (ROS) through a variety of mechanisms, including the inhibition of enzymes involved in ROS production, scavenging, and upregulation or protection of antioxidant defenses [30]. Polyphenols are characterized by a benzene ring that links one or more hydroxyl groups. These compounds are abundant in the plant kingdom and are linked to the health-promoting properties of certain foods, such as vegetables and fruits. They allow growth and reproduction, protect against pathogens and predators, and contribute to the color and sensory properties of fruits and vegetables. Among the many physiological properties of phenolic compounds are anti-allergic, antiatherogenic, anti-inflammatory, antimicrobial, antioxidant, antithrombotic, cardioprotective, and vasodilatory. These properties make polyphenols interesting as food-derived drugs, and epidemiological studies have shown that populations consuming foods rich in specific polyphenols have a lower incidence of cancer or chronic inflammatory diseases [31,32]. As previously stated, *Co ext.* was high in 5-CQA, 5-p-CoQUA, and 3-CQA. They are members of the chlorogenic acids (CGAs) family, which is a large group of plant polyphenols found in the human diet. Epidemiological studies report that the consumption of CGA-rich beverages, such as coffee, tea, wine, various herbal infusions, and some fruit juices, is involved in a lower risk of developing various chronic diseases [22]. CGAs mitigate oxidative stress and the consequent associated negative effects and may also have anti-inflammatory properties by modulating several key metabolic pathways [22]. These literature studies support the findings regarding the anti-inflammatory and antioxidant activities of *Co ext.* reported in this paper.

Because fruit pulp is rarely used as a starting material for in vitro culture to produce secondary metabolites, the findings of this study are difficult to compare to other callus data reported in the literature.

However, this impossibility of comparison does not exclude the importance of the use of callus culture for SMs production. In fact, this propagation technique allows us to obtain products of uniform quality, keep under control the various environmental parameters, avoid the use of chemical synthesis of target compounds and the use of pesticides typical of conventional agriculture, and reduce the amount of water used and the production of waste material. The biodiversity of the various plant species is preserved, and the species themselves are protected from extinction. Furthermore, plant secondary metabolites can be produced in large quantities from small portions of the desired plant material. These aspects make this technique extremely advantageous and applicable in various areas, such as the food industry, pharmaceutical industry, and cosmetics industry [33].

## 5. Conclusions

The results obtained in this study deepen and expand the positive actions of the callus extract of *C. oblonga* Mill. in cell models, where we demonstrated its anti-inflammatory activity and antioxidant properties. The anti-inflammatory effect occurred in both macrophage and keratinocyte cells. A drastic reduction in NO levels was detected in the immune cells, as well as a significant reduction in iNOS and in pro-inflammatory IL-6, IL-1β, ikBα, and ICAM cytokines gene expression in the epidermal cells. The antioxidant effect of the extract was shown as a reduction in ROS levels in oxidatively injured keratinocytes. The limitation of our designed experimental procedure is linked to the cell-model system and, to exceed this limit and confirm these *Co ext*. beneficial effects, further research will be carried out on the *Caenorhabditis elegans* model. The findings presented here could contribute to the implementation of anti-aging non-pharmacologic strategies to extend the health span and prevent age-related diseases in people. The achievement of this last goal is extremely important also considering the impact of older people in socio-economic and public health terms.

## Figures and Tables

**Table 1:** Employed primers for real-time polymerase chain reaction (PCR) analysis.

	Sequence	Accession Number
iNOS	F-5′-TGACCATCATGGACCACCAC-3′ R-5′-ACCAGCCAAATCCAGTCTGC-3′	NM_000625.4
IL6	F-5′-GGTACATCCTCGACGGCATCT-3′ R-5′-GTGCCTCTTTGCTGCTTTCAC-3′	XM_005249745.6
IL-1β	F-5-AAAGAAGAAGATGGAAAAGCGATT-3′ R-5′-GGGAACTGGGCAGACTCAAATTC-3′	XM_047444175
IkBα	F-5′-GCTGCTGATGTCAATGCTCA-3′ R-5′-ACACCAGGTCAGGATTTTGC-3′	NM_020529.3
ICAM	F-5′-CCTTCCTCACCGTGTACTGG-3′ R-5′-AGCGTAGGGTAAGGTTCTTGC-3′	NM_000201
36B4	F 5′-CGACCTGGAAGTCCAACTAC-3′ R 5′- ATCTGCTGCATCTGCTTG-3′	NM_053275.4

## Data Availability

Data available on request from the author.
